# 16S rRNA gene sequencing of mock microbial populations- impact of DNA extraction method, primer choice and sequencing platform

**DOI:** 10.1186/s12866-016-0738-z

**Published:** 2016-06-24

**Authors:** Fiona Fouhy, Adam G. Clooney, Catherine Stanton, Marcus J. Claesson, Paul D. Cotter

**Affiliations:** Teagasc Food Research Centre, Moorepark, Fermoy, Co. Cork Ireland; School of Microbiology, University College Cork, Cork, Ireland; APC Microbiome Institute, University College Cork, Cork, Ireland

**Keywords:** Next-generation sequencing, Mock communities, 16S rRNA, MiSeq, Ion PGM, Gut microbiota, Bias, DNA extraction

## Abstract

**Background:**

Next-generation sequencing platforms have revolutionised our ability to investigate the microbiota composition of complex environments, frequently through 16S rRNA gene sequencing of the bacterial component of the community. Numerous factors, including DNA extraction method, primer sequences and sequencing platform employed, can affect the accuracy of the results achieved. The aim of this study was to determine the impact of these three factors on 16S rRNA gene sequencing results, using mock communities and mock community DNA.

**Results:**

The use of different primer sequences (V4-V5, V1-V2 and V1-V2 degenerate primers) resulted in differences in the genera and species detected. The V4-V5 primers gave the most comparable results across platforms. The three Ion PGM primer sets detected more of the 20 mock community species than the equivalent MiSeq primer sets. Data generated from DNA extracted using the 2 extraction methods were very similar.

**Conclusions:**

Microbiota compositional data differed depending on the primers and sequencing platform that were used. The results demonstrate the risks in comparing data generated using different sequencing approaches and highlight the merits of choosing a standardised approach for sequencing in situations where a comparison across multiple sequencing runs is required.

**Electronic supplementary material:**

The online version of this article (doi:10.1186/s12866-016-0738-z) contains supplementary material, which is available to authorized users.

## Background

The release of the first commercial next-generation sequencer in 2004, the Roche 454 pyrosequencer, resulted in an exponential increase in studies investigating the composition of microbiota in diverse and complex environments. Although Roche 454 platforms were employed in numerous important and enlightening human microbiome studies [1–4], the Illumina MiSeq [5] and Life Technologies Ion PGM [6] platforms are now most commonly used for 16S rRNA gene-based investigations of microbiota composition [7–11]. The decision as to which sequencing platform to utilise for a given study frequently depends on requirements and resources, which vary based on the technology used, the cost/run, data output, amplicon size tolerated, data storage capabilities and error rates.

In order to achieve accurate sequencing results, many factors have to be considered when designing a sequencing study. Numerous studies have investigated the effects of different factors on 16S rRNA gene microbiota data including, in the case of gut microbiota studies, sample type [12] (e.g. faecal vs. cecal), sample storage prior to DNA extraction [13], DNA extraction procedure [14, 15], primers (sequences and 16S rRNA gene regions) [16–18] and the sequencing platform used [19]. This study aims to look at the effects of a combination of 3 factors on sequencing results, namely, 3 different 16S rRNA gene primer sets, use of the Illumina MiSeq and Life Technologies Ion PGM sequencers and comparison of 2 commonly used extraction procedures (QIAamp DNA Stool Mini Kit compared to the repeat bead beating (RBB) method [20] with elements of the Qiagen faecal extraction kit). Regions of the 16S rRNA bacterial gene are most commonly sequenced when using next generation sequencing to study the bacterial composition of an environment. This approach is extremely useful, as even poor quality or low concentrations of DNA can be successfully amplified by degenerate primers and PCR to facilitate sequencing of a region or regions of the 16S rRNA gene, allowing sequencing of diverse populations without prior selection for microbes of interest (as in the case of culture based approaches). However, the particular variable region targeted and primer pair used can impact on the results achieved [21] and the ability of researchers to compare data generated from different sequencing studies. Recent studies have shown the region of the 16S rRNA gene that is sequenced will impact on the results achieved [22].

With respect to the choice of sequencing platform, the two sequencers in question utilise different technologies, which may affect the sequencing results achieved. Briefly, Illumina’s MiSeq is a bench-top version of the HiSeq platform, manufactured by the same company [23]. This platform enables ‘paired-end’ sequencing, is cost effective and can achieve 2 × 300 bp paired read lengths. In contrast, the Ion PGM sequencer utilises semiconductor technology through the real time detection of hydrogen ion concentration [6]. Currently, the Ion PGM produces read lengths of approximately 400 bp in length. As research using high-throughput sequencing continues, there is a need for studies to optimise accuracy while minimising, and where possible eliminating, sequencing bias. While individual studies have compared different primers [17, 21], extraction procedures [14] and sequencing platforms [19], here our aim is to investigate the individual and cumulative effects these 3 factors have on 16S rRNA gene-based investigations of bacterial composition. More specifically, by using a mock community DNA sample and mock community cells for DNA extraction, both with a predetermined composition, we aim to identify which factor(s) have the greatest effects on sequencing results achieved. Thus, our aim was to determine which extraction procedure, region of the 16S rRNA gene and sequencing platform yield results that most accurately reflect the known ratios of bacteria/bacterial DNA in the mock communities. The choice of the V4-V5 and V1-V2 regions to target with our primers was based on the frequency with which they are currently used in such research, thus there is a need to determine which, if either, provides the most accurate results. Our results revealed that the 3 Ion primers detected more of the expected mock communities than was the case when the corresponding MiSeq primers were employed. Ultimately, the choice of PCR primers and sequencing platform had a more notable impact on the results than either of the DNA extraction methods. These results will be of value to researchers when planning future 16S rRNA gene-based microbiota analyses.

## Results

### Sequencing data quality analysis

**Fig. 1:**
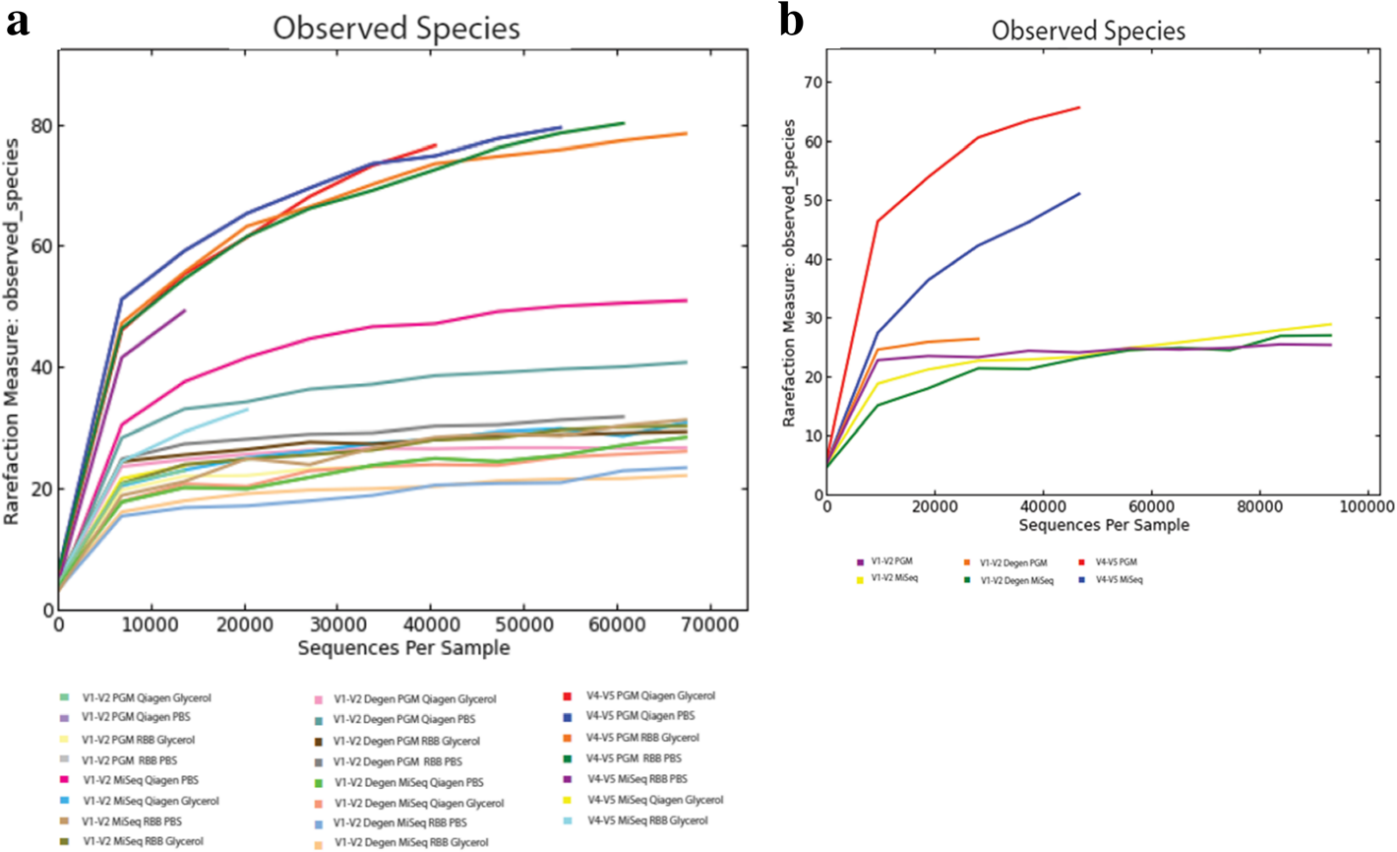
Rarefaction curves based on sample ID and number of observed species for mock cells (**a**) and mock DNA samples (**b**). Curves are approaching or are horizontal with the x axis indicating that additional sequencing would not yield additional novel data

Mock community DNA (HM-782D) and DNA extracted from mock community cells (HM-280/1) was sequenced on the MiSeq and Ion PGM platforms. Details on numbers of sequencing reads, read lengths and percentage of reads retained following quality filtering and chimera removal are provided in Table 1. The percentage of retained reads was similar across platforms and primer sets, with the notable exception of the V4-V5 primers on the Ion PGM, where 80–90 % were retained following chimera removal, compared to an average of 99 % retained for the other primers on both platforms. Rarefaction curves (Fig. 1) demonstrate that the majority of curves begin to plateau, thus additional sequencing is unlikely to yield novel data in most cases.

**Table 1:** Details on number of sequencing reads, read lengths, percentage of reads retained post quality analysis

Primer set	Raw	Quality	Length	Remaining	% Retained	After Chimera Removal	% Chimeras	% Retained
MiSeq								
V4-V5								
Mock DNA	47966	Q25	365–385	42701	89.023475	47966	0	100
Qiagen PBS^a^								
Qiagen glycerol	14071	Q25	365–385	13724	97.533935	13717	0.05100554	99.94899446
RBB PBS	18072	Q25	365–385	17253	95.4681275	18026	0.25453741	99.74546259
RBB glycerol	22650	Q25	365–385	20534	90.6578366	22626	0.10596026	99.89403974
V1-V2								
Mock DNA	576244	Q25	305–325	310254	53.840734	308989	0.40773044	99.59226956
Qiagen PBS	206140	Q25	305–325	165035	80.05966	164117	0.55624564	99.44375436
Qiagen glycerol	274886	Q25	305–325	153566	55.86534	152034	0.99761666	99.00238334
RBB PBS	420677	Q25	305–325	327953	77.958386	324319	1.10808561	98.89191439
RBB glycerol	342405	Q25	305–325	189474	55.336224	181819	4.04013216	95.95986784
V1-V2 deg								
Mock DNA	339219	Q25	305–325	164586	48.5190983	161382	1.94670264	98.05329736
Qiagen PBS	432220	Q25	305–325	170830	39.5238536	166923	2.28706902	97.71293098
Qiagen glycerol	277087	Q25	305–325	100478	36.262257	100031	0.4448735	99.5551265
RBB PBS	407061	Q25	305–325	111020	27.2735536	110057	0.86741128	99.13258872
RBB glycerol	373903	Q25	305–325	117567	31.4431818	116400	0.99262548	99.00737452
Ion PGM								
V4-V5								
Mock DNA	123511	Q25	420–440	57467	46.5278396	51923	9.64727583	90.35272417
Qiagen PBS	173203	Q25	420–440	74942	43.2683037	60366	19.4497078	80.55029223
Qiagen glycerol	194132	Q25	420–440	58267	30.0141141	49474	15.0908748	84.90912523
RBB PBS	211696	Q25	420–440	77227	36.4801413	68006	11.9401246	88.05987543
RBB glycerol	203949	Q25	420–440	84407	41.386327	71316	15.5093772	84.49062282
V1-V2								
Mock DNA	389410	Q25	360–380	191184	49.0958116	190016	0.61092978	99.38907022
Qiagen PBS	21501	Q25	360–380	14852	69.0758569	14804	0.3231888	99.6768112
Qiagen glycerol	35900	Q25	360–380	26518	73.8662953	26418	0.37710235	99.62289765
RBB PBS	35343	Q25	360–380	19157	54.2030954	19046	0.57942267	99.42057733
RBB glycerol	62195	Q25	360–380	42150	67.7707211	42003	0.34875445	99.65124555
V1-V2 deg								
Mock DNA	207570	Q25	360–380	75999	36.6136725	71500	5.91981473	94.08018527
Qiagen PBS	398459	Q25	360–380	236444	59.3396058	231427	2.12185549	97.87814451
Qiagen glycerol	439533	Q25	360–380	214562	48.8159023	208065	3.02802919	96.97197081
RBB PBS	376207	Q25	360–380	180289	47.9228191	174723	3.08726545	96.91273455
RBB glycerol	389283	Q25	360–380	184442	47.3799267	166537	9.70765878	90.29234122

^a^DNA failed to amplify with V4-V5 MiSeq primers for the Qiagen PBS extracted cells so no sequencing data for this extraction sample

### Effects of primers and sequencing platform on mock DNA results

**Fig. 2:**
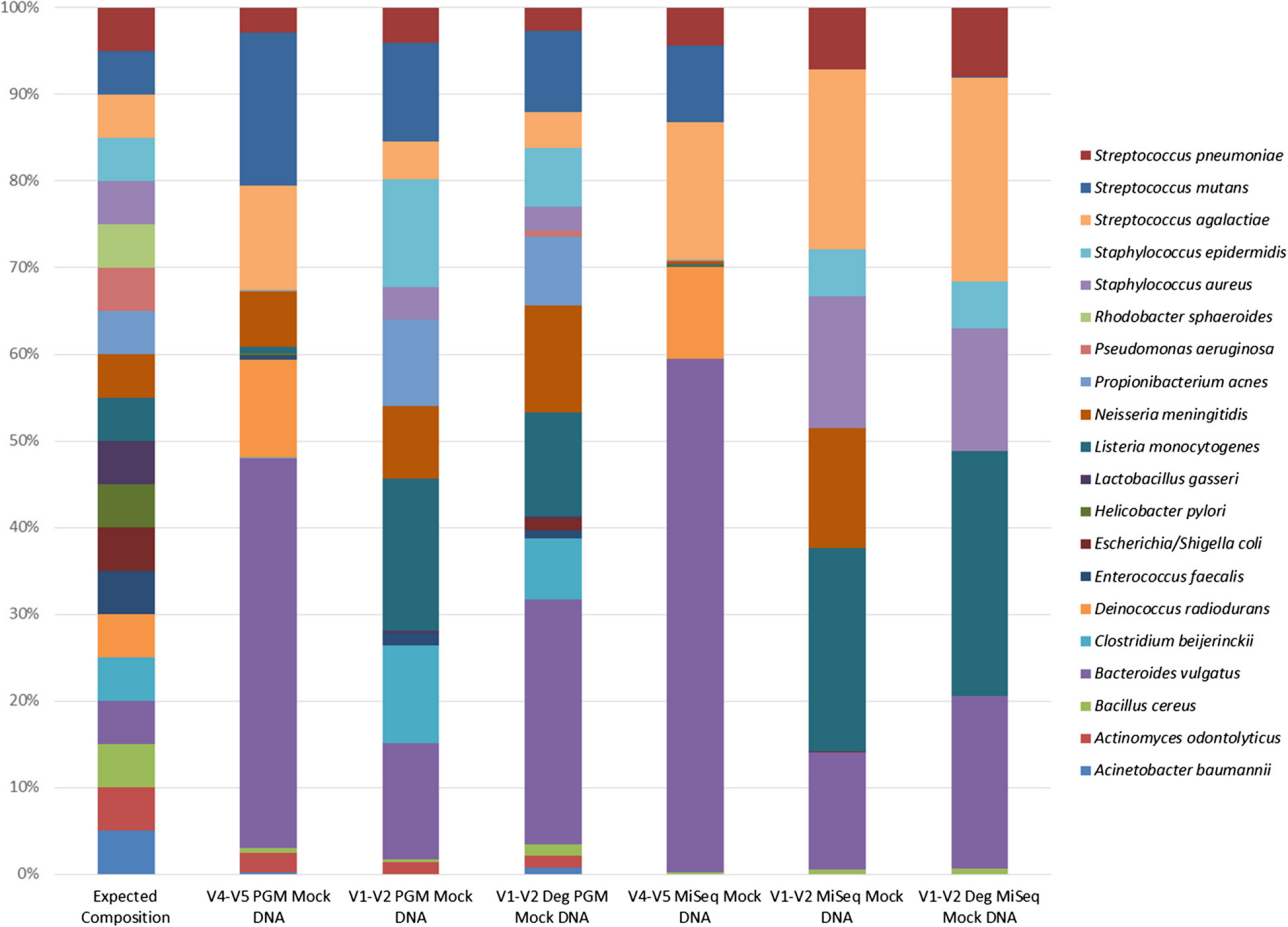
Percentage relative abundance of expected species (*n* = 20) detected in mock community DNA based on sequencing platform and primer set used

It was anticipated that sequencing of the mock DNA (HM-782D) would reveal the presence of 20 species, based on compositional details from the supplier (BEI resources). The 3 primer sets differed in the number of species detected relative to the number of anticipated species present in this DNA sample. The V4-V5 PGM combination was the only combination that detected the template DNA from all of the 20 mock community species (Table 2). In general, the 3 Ion PGM primer sets detected more of the 20 mock community species than the equivalent MiSeq primer sets. There were also a number of instances of mis-identification i.e. where taxa not represented in the mock community DNA were detected. The mis-identified species were, in the majority of cases, closely related to species known to be present in the mock samples (e.g. *E. faecium* detected but *E. faecalis* DNA is present in the mock DNA sample. The SPINGO species classifier highlights that these species share 96.4 % species alignment). Figure 2 more specifically highlights the differences in the data generated. As can be seen in Fig. 2, all primers gave results that differed from those expected for the mock DNA community. The V4-V5 primers gave the most comparable results across platforms while the V1-V2 degenerate primer set used on the Ion PGM platform gave results that most closely matched those expected of an even mock community distribution of 20 species.

**Table 2:** Number of expected vs. detected species in mock DNA and cells

	Expected	Detected	No. of expected species detected	% of expected species detected	% Misidentified/false hit
MiSeq					
V4-V5 mock DNA	20	29	16	80	44
V1-V2 mock DNA	20	37	15	75	59
V1-V2 deg mock DNA	20	34	16	80	53
V4-V5 RBB PBS	22	51	16	73	68
V4-V5 Qiagen glycerol	22	24	17	77	29
V4-V5 RBB glycerol	22	30	16	73	47
V1-V2 Qiagen PBS	22	70	14	64	80
V1-V2 Qiagen glycerol	22	36	15	68	58
V1-V2 RBB PBS	22	40	16	73	60
V1-V2 RBB glycerol	22	36	17	77	53
V1-V2deg Qiagen PBS	22	38	15	68	61
V1-V2deg Qiagen glycerol	22	32	12	55	63
V1-V2deg RBB glycerol	22	29	14	64	52
V1-V2deg RBB PBS	22	31	12	55	61
Ion PGM					
V4-V5 mock DNA	20	33	20	100	40
V1-V2 mock DNA	20	27	19	95	29
V1-V2 deg mock DNA	20	27	19	95	29
V4-V5 Qiagen PBS	22	37	20	91	46
V4-V5 RBB PBS	22	40	20	91	50
V4-V5 Qiagen glycerol	22	31	20	91	35
V4-V5 RBB glycerol	22	38	20	91	47
V1-V2 Qiagen PBS	22	18	17	77	6
V1-V2 Qiagen glycerol	22	30	18	82	40
V1-V2 RBB PBS	22	24	18	82	25
V1-V2 RBB glycerol	22	26	18	82	31
V1-V2 deg Qiagen PBS	22	65	20	91	69
V1-V2 deg Qiagen glycerol	22	28	21	96	25
V1-V2 deg RBB glycerol	22	37	22	100	41
V1-V2deg RBB PBS	22	40	20	91	50

*RBB* repeat bead beating extraction method

Note: DNA failed to amplify with V4-V5 MiSeq primers for the Qiagen PBS extracted cells so no sequencing data for this extraction sample

**Fig. 3:**
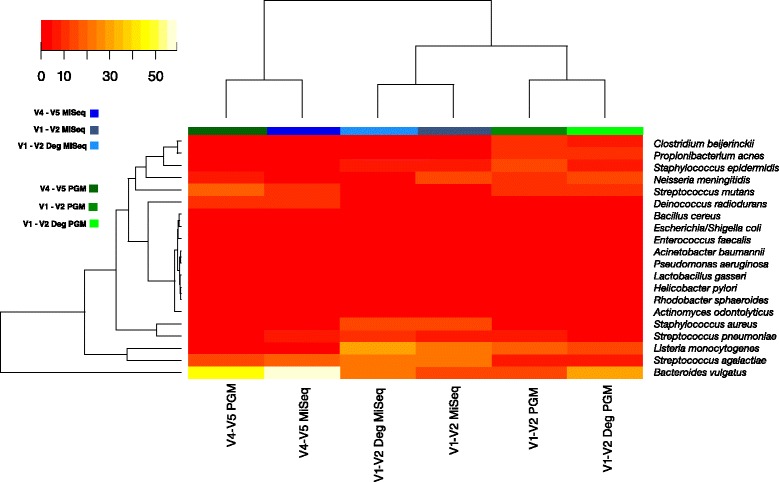
Heat map of species abundance. Only the 20 expected taxa from mock DNA (HM-782D) were included. Hierarchical clustering was performed using hclusing default parameters (complete linkage). The blue colours represent samples sequenced on MiSeq platform while green represent the Ion PGM

Other analyses were carried out to highlight the impact of primer selection and sequencing platform on the outcome of studies. A heat map of taxa abundance (Fig. 3) was generated using Spearman correlations and Ward Clustering. The results highlighted that samples separated based on sequencing platform used, with MiSeq (blue) to the left and Ion PGM (green) to the right. This is with the exception of the MiSeq V4-V5 primer which clusters with the V4-V5 Ion PGM primers. Hence the use of this primer pair is less influenced by platform choice.

### Effects of extraction procedure on sequencing results achieved

**Fig. 4:**
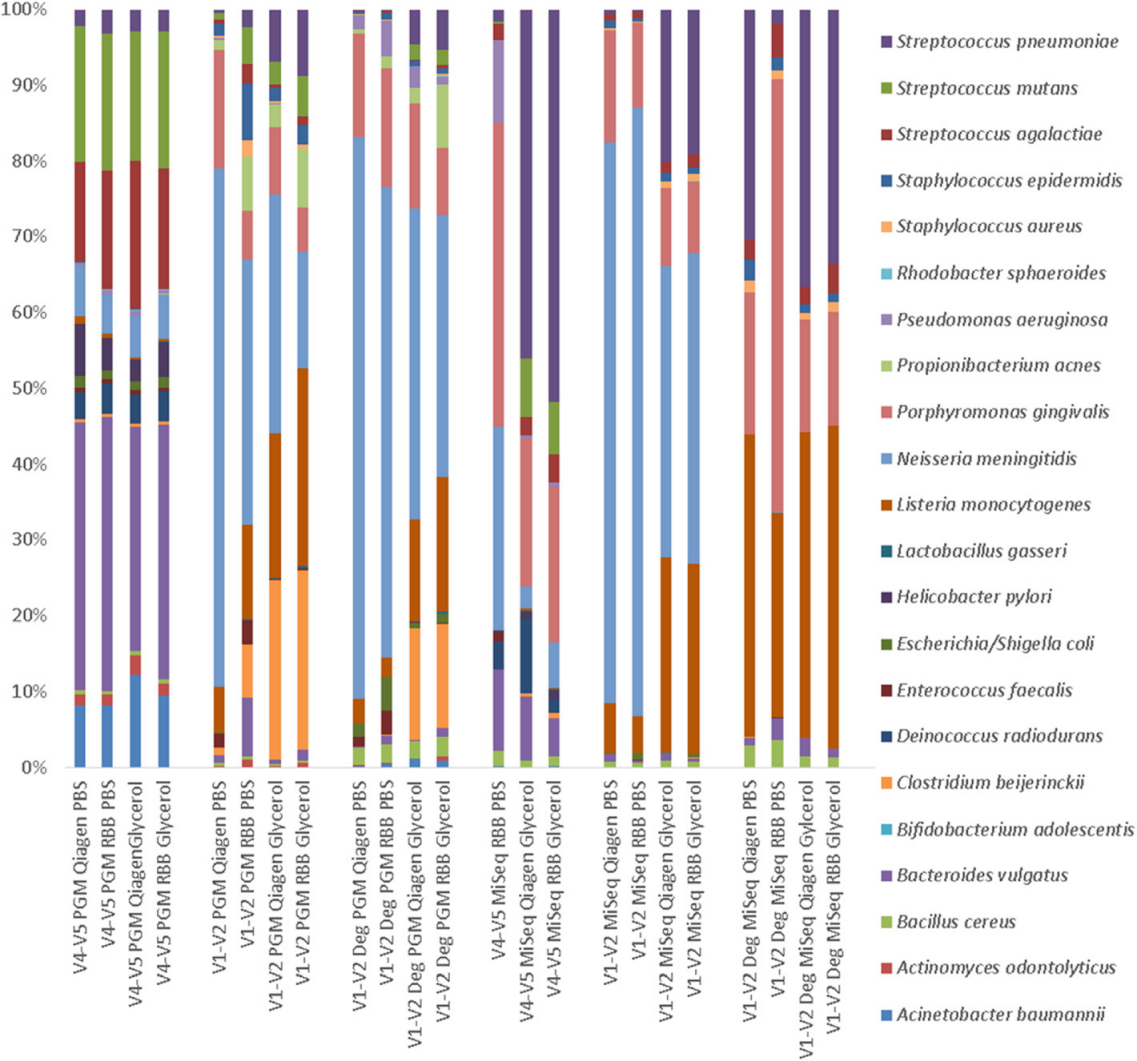
Percentage relative abundance of expected species based on extraction procedure

Having demonstrated the effects of both 16S rRNA gene primer choice and sequencing platform on results, we next aimed to determine the effects of DNA extraction procedures on the sequencing results achieved. As shown in Fig. 4 and Additional file 1, the relative abundances of species detected was more dependent on the primers and platform used, rather than on the choice of extraction procedure. Notable differences occurred based on storage agent (i.e. between glycerol ± PBS), namely the glycerol stocked cells had a higher relative abundance of *Streptococcus, Clostridium* and *Listeria* compared to the PBS + glycerol cells. This was true for sequencing results from both platforms and all primers except using V4-V5 primers on the Ion PGM where similar levels of these bacteria were seen between all extracts. Additionally, V4-V5 MiSeq RBB extracted PBS cells were quite different to either the V4-V5 Qiagen extracted glycerol and RBB glycerol cells. Additionally, the Qiagen PBS extracted DNA failed to amplify with the MiSeq V4-V5 primers, while other primers amplified this DNA. Thus perhaps inhibitors in this sample interacted more strongly with these primers preventing PCR amplification. These results suggest subtle differences occur in sequencing data as a result of sample storage agent and DNA extraction protocol used.

**Fig. 5:**
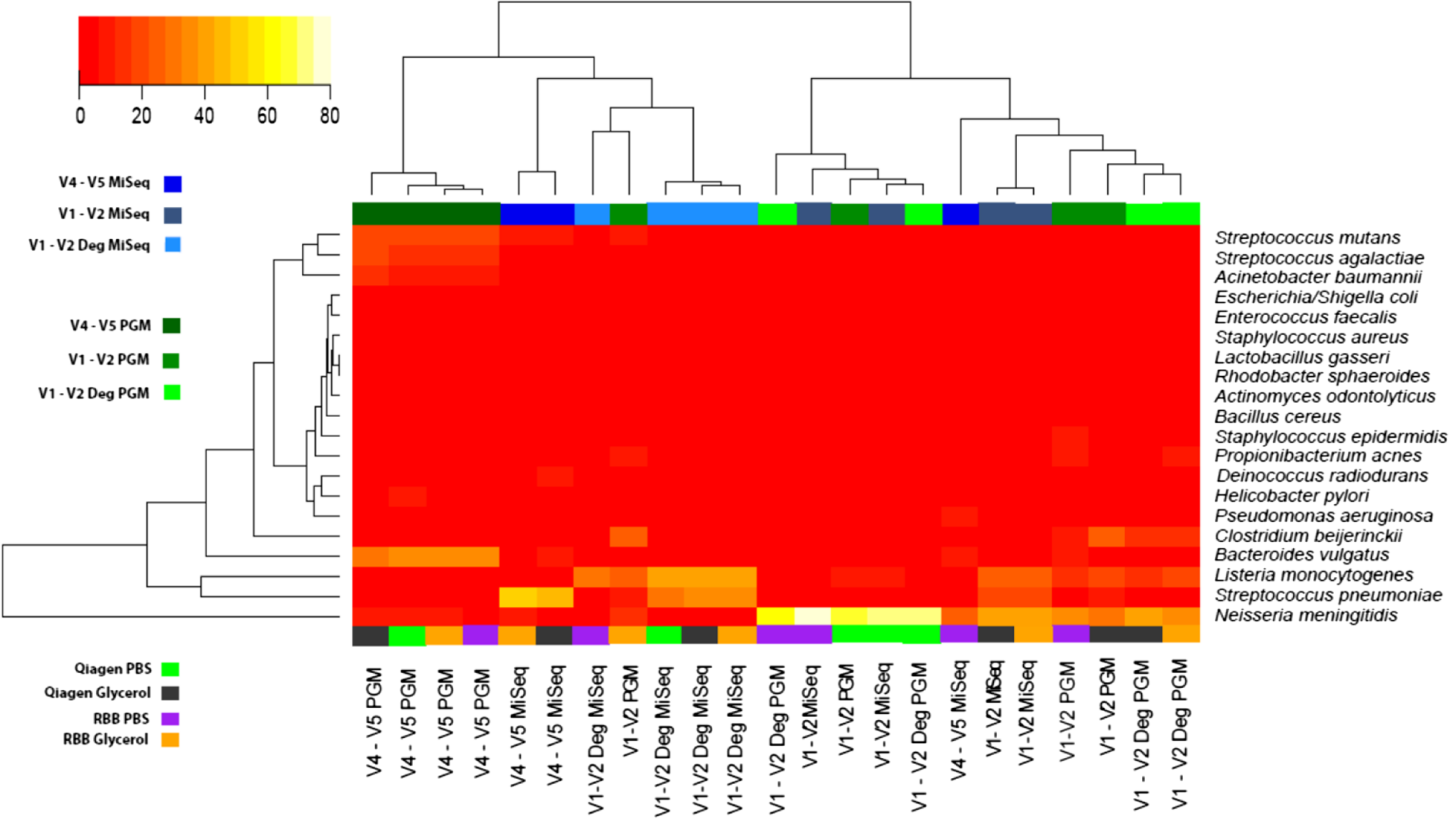
Heat map of species abundance by sequencer and extraction method for mock community cells. Only expected taxa were included and hierarchical clustering was performed using hclust default parameters (complete linkage). The *top* colour legend depicts the sequencing technology and primer set. The *blue* colours represent samples sequenced on MiSeq platform while *green* represent the Ion PGM. The *bottom* legend represents the samples and extraction method

As was seen for the mock DNA, the different primer sets impacted on the species detected in the mock cells. There was a strong impact of primer choice on the results, with samples amplified with the same primers being more similar than those amplified with different primers. Extraction method had a lesser effect on overall composition, with samples extracted using the RBB or the Qiagen method and amplified with the same primer, yielding similar results. Additionally, as shown in Fig. 5, the samples do not show clustering based on extraction method, with samples extracted using different extraction procedures, but amplified with the same primers yielding similar results.

We anticipated that 22 species would be detected from the DNA extracted from the mock community cells, however, bioinformatic analysis again indicated the presence of species known not to be within the mock community. None of the primer sets, when used on the MiSeq platform (irrespective of extraction method or storage agent), detected all 22 expected species (Table 2). Indeed the best performing primer sets only detected 77 % of the expected species (V4-V5 Qiagen glycerol and V1-V2 RBB glycerol extracts). All primer pairs used with the Ion PGM platform detected a greater percentage of expected species (77–100 %) compared to the corresponding primers used on the MiSeq (55–77 %). The V1-V2 degenerate Ion PGM primers used on the RBB glycerol extracted cells detected 100 % of the expected species.

The heat map for the mock cells gave similar results as for the mock community DNA (Fig. 5). The V4-V5 amplified samples cluster together irrespective of extraction procedure or sequencing platform used, with the exception of the RBB PBS V4-V5 MiSeq sample that clustered with the V1-V2 amplified samples. Observing the coloured line indicating the extraction method, it is evident that there is clustering by primer set used and not by extraction method. The heat map also shows how species abundances differed across samples with primer choice, rather than extraction method, appearing to cause differences in species detected between samples.

## Discussion

With the rapid increase in studies investigating the microbiota of diverse environments using high-throughput sequencing approaches, it is critical that the impact of numerous factors on the sequencing results be determined. This study analysed the effects of DNA extraction procedures, 16S rRNA gene primer design and the choice of sequencing platform on outcomes using mock community cells and DNA.

The vast majority of sequencing studies have relied on sequencing of the 16S rRNA gene to determine the bacteria present in an environment [1, 3]. Previous studies have also examined the effects of primers on sequencing outcomes by amplifying the V1-V3, V3-V5 and V6-V9 regions of the 16S rRNA gene from the same mock community cells as used in this study (HM 280/1) and sequencing using Sanger and 454-pyrosequencing platforms [16]. They also noted the effect of the region of the 16S rRNA gene targeted on sequencing data. Our study used primers targeting the V4-V5 and V1-V2 regions and employed the Illumina MiSeq and Ion PGM platforms. Despite both studies using the same mock community cells (HM 280/1), differences occurred between our data sets, likely due to a combination of primer and sequencer effects. Similar to the study by Haas et al., our study also noted non-uniform relative abundances in the mock communities. The results demonstrated that the V4-V5 primers gave the most comparable results across platforms, which could be of benefit to researchers moving between newer sequencing platforms. However, this result must also be considered in light of the fact that the same primer sets gave skewed abundances. Thus while this primer set gave the most comparable results across platforms suggesting it is least affected of the primers sets by platform used, the results still show discrepancies between the anticipated and achieved results with this primer set. The results from the V1-V2 and V1-V2 degenerate primers were distinct from the V4-V5 primers and differed in their detection of species. Considerable differences occurred based on which primer and platform was used. Only two combinations, namely the V4-V5 Ion PGM primers used on the mock DNA and the V1-V2 degenerate Ion PGM primers used on the RBB glycerol cells, detected 100 % of the expected species. It is worth noting that the same primers used on other extracted or mock DNA templates did not detect 100 % of the expected species, thereby again highlighting that the results are due to a combination of factors, including DNA extraction procedure, primer choice and sequencing platform. Furthermore, no primer set detected the expected species exclusively and all gave false hits (% of reads achieved that were not expected relative to total reads achieved) (varying from 6 % for the Ion PGM V1-V2 Qiagen PBS sample to 69 % for the Ion PGM V1-V2 degenerate Qiagen PBS sample). These were present at very low relative abundances and were closely related to the actual species present in the mock communities, thus we suggest they were mis-assigned at species level, due to similarities in their 16S rRNA gene sequence e.g. *E. faecalis* present in the mock community but *E. faecium* also assigned at species level. Based on these findings it appears that the primers consistently performed best on the Ion PGM platform, with higher percentages of expected species detected and lower false hits compared to the MiSeq platform. A recent study also took a similar approach to ours and compared the results of a mock community sequenced using primers targeting the V1-V2 region and sequenced on the MiSeq and Ion PGM platforms [19]. The study found the relative abundances to be generally in agreement with the expected community composition and the results to be similar across platforms. While our study did not analyse replicates (due to limitations in starting material), Salipante et al. did not find significant differences between replicates, which is consistent with previous findings also [24]. Our findings are similar to those of Tremblay et al. [22] who also showed differences in sequencing results on the 454-pyrosequencer and the MiSeq when different regions of the 16S rRNA gene were targeted, using a mock microbial community. In this instance, the authors compared the V4, V6-V8 and V7-V8 regions and found that the V4 primers gave the least biased results. We also found the V4-V5 primer set to yield the most comparable results across platforms. The authors also highlight that currently there is no consensus on which primer set yields the best result; therefore they suggest the potential to use shotgun metagenomics to interrogate your dataset and to compare the results with that of your different primer sets under investigation. However, due to cost this is still not a feasible approach for most studies but could be used perhaps to select between primer sets prior to commencing a series of sequencing-based studies.

This study also conducted a direct comparison of the MiSeq and Ion PGM platforms, both of which are being used increasingly for 16S rRNA amplicon sequencing studies. The results indicated that not only the depth of sequencing achieved differs by platform, but also the percentage of retained sequences following quality filtering and chimera removal. We found the lowest percentage of reads was retained from the V4-V5 primer sequences from the Ion PGM (80–90 % retained following chimera removal compared to an average of 99 % retained for the other primers on both platforms). This may be due to the fact that these were the longest reads achieved on the Ion PGM at 380–400 bp. Currently 400 bp is the longest read length supported by this platform and although we achieved longer read lengths with this primer set, the quality of these reads was considerably lower than the shorter reads with the V1-V2 primer sets (335–355 bp), resulting in increased numbers of reads being removed during quality filtering and chimera removal. This study has clearly shown the impact of sequencing platform on the results achieved, a finding also observed in a previous study [22] which showed that samples clustered by sequencing platform used. A recent study also compared the MiSeq and Ion PGM platforms for sequencing a mock microbial community using V1-V2 16S rRNA primers [19]. Our results are in agreement with this previous study that both platforms offer good sequencing depth and are a good alternative to older platforms. However, they noted that more studies looking at different regions of the 16S rRNA gene were needed to fully comprehend the impact these factors have on sequencing outcomes. This previous study also highlighted the potential to minimise sequencing artifacts using bidirectional sequencing and also through optimization of flow order on the Ion PGM platform. Again this study did not conclude as to which platform/primer combination gave the best results. Thus, we conclude that based on ours and previous data, the most suitable primer and platform to use for sequencing studies remains unclear. Perhaps the inclusion of mock communities or the comparison of 16S rRNA based data to shotgun metagenomic data may enable an optimised approach to be devised at the beginning of a large sequencing-based trial and there after the use of this optimised approach would limit variation between results within this trial. Thus we share the conclusions of Tremblay et al. [22] that based on all current knowledge, protocol consistency remains more pertinent to the study outcome than primer or sequencing platform choice.

DNA extraction procedure has a significant impact on sequencing results [25, 26]. Several studies have previously shown the effects of using different commercial kits for DNA extraction from faecal samples on sequencing outcomes [14, 15, 17]. Our approach was to focus specifically on just two extraction methods commonly used in microbiota studies to establish if the widely used Qiagen DNA extraction approach was as successful as the RBB approach or if the additional bead beating steps yielded more accurate results. Both extraction procedures yielded DNA that gave comparable results with respect to phylogeny. This may be due to the similarity in these approaches, while the use of a different commercial DNA extraction kit could yield significantly different results. Additionally, this study used mock community cells to investigate the effects of DNA extraction procedure. This is a relatively simple microbiota community relative to, e.g. faecal samples. Thus results suggesting that the extraction method has minimal effects on microbiota sequencing data could in fact be due to the ease of extraction of DNA from the mock community cells. While we have shown both DNA extraction procedures to yield similar sequencing results in this instance, it is our recommendation that the selection and use of just one DNA extraction method for longitudinal studies is vital to ensure differences in the data that may be observed are not occurring due to extraction bias.

## Conclusions

This study provides a direct comparison of the Illumina MiSeq and Ion PGM sequencers and has shown that the MiSeq and Ion PGM sequencers offer good sequencing depth and provides information at species level, not attainable using older platforms. Given the demonstrated differences in microbiota composition due to primer choice and sequencing platform used, the need for the use of internal controls on sequencing runs is evident. Overall, our results are significant as they highlight important considerations for designing and interpreting sequencing studies. Thus as we enter an era of rapid sequencing development, advancement and improvement, it is of utmost importance to carefully consider, assess and continually review best practice regarding designing, conducting and interpreting microbiota sequencing studies.

## Methods

### PCR primers for 16S rRNA gene sequencing

PCR primers for 16S rRNA gene sequencing using the Illumina MiSeq sequencing platform were designed to consist of an Illumina adaptor sequence, a 12 nt index (barcode) sequence, a 10 nt primer pad region, a 2 nt linker region and the gene specific primer sequence (Table 3). Three primer sets, one targeting the V4-V5 region [23] and two primer pairs targeting the V1-V2 region of the 16S rRNA gene [4], with primer set 2 using a degenerate forward primer [17] were used for sequencing to determine the effect of primer design and the region of the 16S rRNA gene which is targeted, on the sequencing outcomes. A corresponding set of 3 primer pairs were generated for use on the Ion PGM platform and were designed to contain the Ion PGM linker sequence, a unique 10 nt Golay barcode sequence, a 2 nt spacer sequence and the gene specific sequence (Table 4).

**Table 3:** Sequences of primers used for MiSeq sequencing

Sample	Primer sequence	Barcode	Ref
V4-V5 primer			[23]
Forward primer	AATGATACGGCGACCACCGAGATCTACACTATGGTAATTGGGTGCCAGCMGCCGCGGTAA		
Read 1 primer	TATGGTAATTGGGTGCCAGCMGCCGCGGTAA		
Read 2 primer	AGTCAGTCAGTTCCGTCAATTYYTTTRAGTTT		
Index primer	AAACTYAAARRAATTGACGGAACTGACTGACT		
Reverse barcoded primers			
PBS Qiagen	CAAGCAGAAGACGGCATACGAGATTAACGTGTGTGCAGTCAGTCAGTTCCGTCAATTYYTTTRAGTTT	TAACGTGTGTGC	
PBS RBB	CAAGCAGAAGACGGCATACGAGATCATTATGGCGTGAGTCAGTCAGTTCCGTCAATTYYTTTRAGTTT	CATTATGGCGTG	
Qiagen Glycerol	CAAGCAGAAGACGGCATACGAGATCCAATACGCCTGAGTCAGTCAGTTCCGTCAATTYYTTTRAGTTT	CCAATACGCCTG	
RBB Glycerol	CAAGCAGAAGACGGCATACGAGATGATCTGCGATCCAGTCAGTCAGTTCCGTCAATTYYTTTRAGTTT	GATCTGCGATCC	
Mock DNA	CAAGCAGAAGACGGCATACGAGATCAGCTCATCAGCAGTCAGTCAGTTCCGTCAATTYYTTTRAGTTT	CAGCTCATCAGC	
V1-V2 set 1			[4]
Forward primer	AATGATACGGCGACCACCGAGATCTACACTATGGTAATTTCAGAGTTTGATCCTGGCTCAG		
Read 1 primer	TATGGTAATTTCAGAGTTTGATCCTGGCTCAG		
Read 2 primer	AGTCAGTCAGCATGCTGCCTCCCGTAGGAGT		
Index primer	ACTCCTACGGGAGGCAGCATGCTGACTGACT		
Reverse barcoded primers			
PBS Qiagen	CAAGCAGAAGACGGCATACGAGATTCTTGGAGGTCAAGTCAGTCAGCATGCTGCCTCCCGTAGGAGT	TCTTGGAGGTCA	
PBS RBB	CAAGCAGAAGACGGCATACGAGATTCACCTCCTTGTAGTCAGTCAGCATGCTGCCTCCCGTAGGAGT	TCACCTCCTTGT	
Qiagen Glycerol	CAAGCAGAAGACGGCATACGAGATGCACACCTGATAAGTCAGTCAGCATGCTGCCTCCCGTAGGAGT	GCACACCTGATA	
RBB Glycerol	CAAGCAGAAGACGGCATACGAGATGCGACAATTACAAGTCAGTCAGCATGCTGCCTCCCGTAGGAGT	GCGACAATTACA	
Mock DNA	CAAGCAGAAGACGGCATACGAGATTCATGCTCCATTAGTCAGTCAGCATGCTGCCTCCCGTAGGAGT	TCATGCTCCATT	
V1-V2 degenerate			[17]
Forward primer	AATGATACGGCGACCACCGAGATCTACACTATGGTAATTTCAGMGTTYGATYMTGGCTCAG		
Read 1 primer	TATGGTAATTTCAGMGTTYGATYMTGGCTCAG		
Read 2 primer	AGTCAGTCAGCATGCTGCCTCCCGTAGGAGT		
Index primer	ACTCCTACGGGAGGCAGCATGCTGACTGACT		
Reverse barcoded primers			
PBS Qiagen	CAAGCAGAAGACGGCATACGAGATTCTTGGAGGTCAAGTCAGTCAGCATGCTGCCTCCCGTAGGAGT	TCTTGGAGGTCA	
PBS RBB	CAAGCAGAAGACGGCATACGAGATTCACCTCCTTGTAGTCAGTCAGCATGCTGCCTCCCGTAGGAGT	TCACCTCCTTGT	
Qiagen Glycerol	CAAGCAGAAGACGGCATACGAGATGCACACCTGATAAGTCAGTCAGCATGCTGCCTCCCGTAGGAGT	GCACACCTGATA	
RBB Glycerol	CAAGCAGAAGACGGCATACGAGATGCGACAATTACAAGTCAGTCAGCATGCTGCCTCCCGTAGGAGT	GCGACAATTACA	
Mock DNA	CAAGCAGAAGACGGCATACGAGATTCATGCTCCATTAGTCAGTCAGCATGCTGCCTCCCGTAGGAGT	TCATGCTCCATT

**Table 4:** Primers for amplification of DNA for sequencing on the Ion PGM platform

	Ion Linker	Barcode	Spacer	Primer	Ref
V4-V5					[23]
Forward barcoded primers					
Mock DNA	CCATCTCATCCCTGCGTGTCTCCGACTCAG	TCCCTTGTCTCC	GT	GTGCCAGCMGCCGCGGTAA	
PBS Qiagen	CCATCTCATCCCTGCGTGTCTCCGACTCAG	ACGAGACTGATT	GT	GTGCCAGCMGCCGCGGTAA	
PBS RBB	CCATCTCATCCCTGCGTGTCTCCGACTCAG	GCTGTACGGATT	GT	GTGCCAGCMGCCGCGGTAA	
Glycerol Qiagen	CCATCTCATCCCTGCGTGTCTCCGACTCAG	ATCACCAGGTGT	GT	GTGCCAGCMGCCGCGGTAA	
Glycerol RBB	CCATCTCATCCCTGCGTGTCTCCGACTCAG	TGGTCAACGATA	GT	GTGCCAGCMGCCGCGGTAA	
Reverse primer	CCTCTCTATGGGCAGTCGGTGAT		CC	CCGTCAATTYYTTTRAGTTT	
V1-V2 set 1					[4]
Forward barcoded primers					
Mock DNA	CCATCTCATCCCTGCGTGTCTCCGACTCAG	TGCATACACTGG	GT	AGAGTTTGATCCTGGCTCAG	
PBS Qiagen	CCATCTCATCCCTGCGTGTCTCCGACTCAG	AGTCGAACGAGG	GT	AGAGTTTGATCCTGGCTCAG	
PBS RBB	CCATCTCATCCCTGCGTGTCTCCGACTCAG	ACCAGTGACTCA	GT	AGAGTTTGATCCTGGCTCAG	
Glycerol Qiagen	CCATCTCATCCCTGCGTGTCTCCGACTCAG	GAATACCAAGTC	GT	AGAGTTTGATCCTGGCTCAG	
Glycerol RBB	CCATCTCATCCCTGCGTGTCTCCGACTCAG	GTAGATCGTGTA	GT	AGAGTTTGATCCTGGCTCAG	
Reverse primer	CCTCTCTATGGGCAGTCGGTGAT		CC	TGCTGCCTCCCGTAGGAGT	
V1-V2 set 2					[17]
Forward barcoded primers					
Mock DNA	CCATCTCATCCCTGCGTGTCTCCGACTCAG	GCGATATATCGC	GT	AGMGTTYGATYMTGGCTCAG	
PBS Qiagen	CCATCTCATCCCTGCGTGTCTCCGACTCAG	CGAGCAATCCTA	GT	AGMGTTYGATYMTGGCTCAG	
PBS RBB	CCATCTCATCCCTGCGTGTCTCCGACTCAG	AGTCGTGCACAT	GT	AGMGTTYGATYMTGGCTCAG	
Glycerol Qiagen	CCATCTCATCCCTGCGTGTCTCCGACTCAG	GTATCTGCGCGT	GT	AGMGTTYGATYMTGGCTCAG	
Glycerol RBB	CCATCTCATCCCTGCGTGTCTCCGACTCAG	CGAGGGAAAGTC	GT	AGMGTTYGATYMTGGCTCAG	
Reverse primer	CCTCTCTATGGGCAGTCGGTGAT		CC	TGCTGCCTCCCGTAGGAGT	

### Mock community DNA

To determine the effects of different primer sets, and different DNA extraction procedures on sequencing results, genomic DNA from Microbial Mock Community B (Even, Low Concentration), v5.1L, for 16S rRNA Gene Sequencing (HM-782D), and cells from Microbial Mock Community C in phosphate buffered saline (PBS) (HM-280) and in PBS and 40 % Glycerol (HM-281) were obtained through BEI Resources, NIAID, NIH as part of the Human Microbiome Project (Manassas, VA). Mock community DNA was used as template DNA for sequencing using 3 primer sets, per platform, as outlined below.

### Metagenomic DNA extraction for PCR reactions

Mock community cells (HM-280/1) were used to ascertain the effects of extraction procedure on the sequencing results achieved, thus DNA was extracted from these cells using 2 DNA extraction procedures and DNA was subsequently amplified using 3 Illumina MiSeq and 3 Ion PGM primer sets. DNA was also extracted from mock community cells in PBS (HM-280) and those in PBS + glycerol (HM-281), thus determining if the storage agent of the cells prior to extraction has any effect on the results achieved. DNA was extracted from mock community cells, using previously described methods [2, 27]. Briefly, DNA was extracted from mock community cells (HM-280/1) using a QIAamp DNA Stool Mini Kit (Qiagen, Sussex, UK), with the addition of an initial bead beating step. DNA was also extracted using a RBB approach and a modified Qiagen DNA extraction procedure [13, 20]. Briefly, 1 ml of lysis buffer (500 mM NaCl, 50 mM Tris–HCl pH8.0, 50 mM EDTA and 4 % sodium dodecyl sulphate) was added to the bead beating tubes containing the mock community cell sample. Samples were homogenised for 3 mins at max speed using the Mini Bead beater. Samples were incubated at 70 °C for 15mins. Following centrifugation the supernatant was removed and the bead beating steps repeated. Following pooling of the supernatant, samples were treated with 10 M ammonium acetate (Sigma Aldrich, Ireland), the DNA was pelleted and washed with 70 % ethanol. The DNA was then RNAse and proteinase K treated. Finally the DNA was washed using buffers AW1 and AW2 (QIAmp Fast DNA Stool Mini kit) and eluted in 200 μl of ATE buffer (QIAmp Fast DNA Stool Mini kit).

### PCR amplification and preparation for next generation 16S rRNA gene sequencing

PCR reactions contained 25 μl Biomix Red (MyBio, Kilkenny, Ireland), 1 μl forward primer (Sigma Aldrich, Dublin, Ireland) (10pmol), 1 μl reverse primer (Sigma Aldrich) (10pmol), template DNA (64 ng) and PCR grade water (MyBio). PCR conditions were as follows: V4-V5 primer set: heated lid 110 °C, 94 °C × 3mins, followed by 35 cycles of 94 °C × 45 s, 67 °C × 1 min, 72 °C × 1 min, followed by 72 °C × 2mins and held at 4 °C. For V1-V2 primer set 1: heated lid 110 °C, 94 °C × 2mins, followed by 25 cycles of 94 °C × 1 min, 67 °C × 45 s, 72 °C × 1 min, followed by 72 °C × 2mins and held at 4 °C. Twenty five cycles was chosen, as higher cycle numbers gave non-specific bands. For V1-V2 degenerate primer set 2: heated lid 110 °C, 94 °C × 2mins, followed by 35 cycles of 94 °C × 1 min, 64 °C × 45 s, 72 °C × 1 min, followed by 72 °C × 2mins and held at 4 °C. All PCR reactions were completed in triplicate. Negative controls, where DNA was replaced by PCR grade water, were run for each primer set, with no amplification occurring. Triplicate PCR products were pooled and cleaned using AMPure magnetic bead-based purification system (Beckman Coulter, UK). Cleaned samples were quantified using Picogreen Quant-iT quantification and the Nanodrop 3300 (Fisher Scientific, Dublin, Ireland). To confirm purity and primer specificity of the PCR reactions, samples were analysed using the Agilent Bioanalyser. Samples were subsequently pooled in an equimolar concentration and prepared for sequencing using standard protocols. For MiSeq sequencing, libraries were mixed with Illumina generated PhiX control libraries (20 % of 12.5pM solution) and were denatured using freshly prepared NaOH. Samples were loaded at 6pM and sequenced using a V3 600 cycle kit and our specific 16S rRNA gene sequencing primers. For PGM sequencing, libraries were pooled and loaded at 40pM and were sequenced according to Ion PGM protocols using the Ion 318 v2 chip and the Ion PGM Sequencing 400 kit. Loading concentrations for the respective libraries were as per manufacturer’s recommendations.

### Bioinformatic analysis

Reads for the MiSeq were merged using the QIIME (version 1.8) script *join_paired_ends.py* and the *fastq-join* method [28], however this was not required for PGM reads as they were single-ended. The QIIME script *split_libraries.py* was used to demultiplex both MiSeq and PGM reads with default parameters, however, only reads matching the main length distribution; MiSeq: V1-V2 primers (305–325 bp), V4-V5 primer (365–385 bp) and PGM: V1-V2 primers (335–355 bp), and V4-V5 primer (380–400 bp) and reads with a minimum average quality score of Q25 were retained. Chimeric sequences were removed via USEARCH version 7.0.1090 using the *uchime_ref.py* script and the ChimeraSlayer GOLD database [29]. The Mothur implementation of the Ribosomal Database Project (RDP) classifier was used to assign taxonomy from phylum to genus [30] with a bootstrap cut-off of 50 %. Any sequences outside this cut-off were assigned as unclassified at that particular rank.

Species classification along with *Clostridium* Cluster classification was obtained by utilising the species classifier SPINGO version 1.2 with default parameters [31]. The quality filtered sequence reads for each technology and primer set were inputted into SPINGO and the results were summarised using the script *spingo_summary.py* included with the software. Heat maps were generated in R version 3.1.3. The function heatmap.2 was performed on the mock cell and mock DNA samples with only the expected species included. Hierarchical clustering was conducted using hclust.

## Abbreviations

PBS, phosphate buffered saline; RBB, repeat bead beating; RDP, ribosomal database project.

## Additional file

Additional file 1: Table S1. Percentage relative abundance of expected species detected in the mock cell DNA. (DOCX 21 kb)
