# Supplementary material for: 16S rRNA gene sequencing of mock microbial populations- impact of DNA extraction method, primer choice and sequencing platform
Source: BMC Microbiol. 2016 Jun 24;16:123. doi: 10.1186/s12866-016-0738-z (PMC4921037; doi:10.1186/s12866-016-0738-z)
Supplement: Additional file 1: Table S1. — Percentage relative abundance of expected species detected in the mock cell DNA. (DOCX 21 kb) [file 12866_2016_738_MOESM1_ESM.docx]

**16S rRNA gene sequencing of mock microbial populations; impact of DNA extraction method, primer choice and sequencing platform**

**Running title:** Sequencing of mock microbial populations

Fiona Fouhy^1#^, Adam G. Clooney^2,3#^, Catherine Stanton^1,3^, Marcus J. Claesson^2,3*^ Paul D. Cotter^1,3*^

Additional file 1: Table S1: Percentage relative abundance of expected species detected in the mock cell DNA

|  | *Acinetobacter baumannii* | *Actinomyces odontolyticus* | *Bacillus cereus* | *Bacteroides vulgatus* | *Bifidobacterium adolescentis* | *Clostridium beijerinckii* | *Deinococcus radiodurans* | *Enterococcus faecalis* | *Escherichia/Shigella coli* | *Helicobacter pylori* | *Lactobacillus gasseri* | *Listeria monocytogenes* | *Neisseria meningitidis* | *Porphyromonas gingivalis* | *Propionibacterium acnes* | *Pseudomonas aeruginosa* | *Rhodobacter sphaeroides* | *Staphylococcus aureus* | *Staphylococcus epidermidis* | *Streptococcus agalactiae* | *Streptococcus mutans* | *Streptococcus pneumoniae* |
| --- | --- | --- | --- | --- | --- | --- | --- | --- | --- | --- | --- | --- | --- | --- | --- | --- | --- | --- | --- | --- | --- | --- |
| V4-V5 PGM Qiagen PBS | 8.2470 | 1.3389 | 0.5895 | 35.2225 | 0.0000 | 0.4323 | 3.5200 | 0.5025 | 1.5775 | 6.8800 | 0.0056 | 0.9684 | 6.6611 | 0.0000 | 0.0281 | 0.3228 | 0.1039 | 0.0196 | 0.0589 | 13.1761 | 17.8358 | 2.1614 |
| V4-V5 PGM RBB PBS | 8.1357 | 1.4750 | 0.4754 | 35.9631 | 0.0000 | 0.5047 | 3.9545 | 0.5778 | 1.2068 | 4.2690 | 0.0195 | 0.4535 | 5.2295 | 0.0000 | 0.0414 | 0.5461 | 0.0561 | 0.0293 | 0.0585 | 15.5033 | 18.0584 | 3.1548 |
| V4-V5 PGM Qiagen Glycerol | 12.1654 | 2.6803 | 0.4900 | 29.4967 | 0.0000 | 0.4316 | 3.9134 | 0.5322 | 1.1357 | 2.8393 | 0.0260 | 0.2499 | 5.4126 | 0.0000 | 0.0681 | 0.7009 | 0.1363 | 0.0422 | 0.0422 | 19.5541 | 17.0166 | 2.8588 |
| V4-V5 PGM RBB Glycerol | 9.4490 | 1.6624 | 0.4929 | 33.5201 | 0.0000 | 0.4394 | 3.9293 | 0.4883 | 1.3671 | 4.6431 | 0.0186 | 0.4208 | 5.8475 | 0.0000 | 0.0209 | 0.4952 | 0.0837 | 0.0233 | 0.0326 | 15.9033 | 18.0260 | 2.8110 |
|  |  |  |  |  |  |  |  |  |  |  |  |  |  |  |  |  |  |  |  |  |  |  |
| V1-V2 PGM Qiagen PBS | 0.0085 | 0.1781 | 0.3900 | 1.0344 | 0.0000 | 0.9581 | 0.0000 | 1.9332 | 0.1102 | 0.0000 | 0.0424 | 6.0370 | 68.3144 | 15.6012 | 1.2973 | 0.2968 | 0.0000 | 0.2968 | 1.6873 | 0.4918 | 0.9242 | 0.3900 |
| V1-V2 PGM RBB PBS | 0.0136 | 1.0587 | 0.4547 | 7.6824 | 0.0000 | 7.0987 | 0.0000 | 3.0404 | 0.0271 | 0.0000 | 0.2104 | 12.4466 | 34.9847 | 6.4133 | 7.1938 | 0.0204 | 0.0000 | 2.0835 | 7.4109 | 2.6671 | 4.7777 | 2.3549 |
| V1-V2 PGM Qiagen Glycerol | 0.0157 | 0.1879 | 0.2453 | 0.6262 | 0.0000 | 23.5884 | 0.0000 | 0.1670 | 0.0000 | 0.0052 | 0.0887 | 19.0533 | 31.5103 | 8.9030 | 3.1051 | 0.0731 | 0.0000 | 0.3757 | 1.6648 | 0.4436 | 2.9694 | 6.8991 |
| V1-V2 PGM RBB Glycerol | 0.0140 | 0.5928 | 0.2736 | 1.4663 | 0.0000 | 23.6012 | 0.0000 | 0.2912 | 0.0175 | 0.0000 | 0.3087 | 26.0252 | 15.4208 | 5.8477 | 7.6999 | 0.0456 | 0.0000 | 0.6244 | 2.5011 | 1.1190 | 5.3496 | 8.7452 |
|  |  |  |  |  |  |  |  |  |  |  |  |  |  |  |  |  |  |  |  |  |  |  |
| V1-V2 Deg PGM Qiagen PBS | 0.2213 | 0.0511 | 2.3424 | 0.0883 | 0.0074 | 0.1275 | 0.0025 | 1.2824 | 1.7186 | 0.0000 | 0.0045 | 3.1930 | 74.1429 | 13.5362 | 0.5727 | 1.9305 | 0.0000 | 0.0506 | 0.3553 | 0.0963 | 0.0377 | 0.1310 |
| V1-V2 Deg PGM RBB PBS | 0.4127 | 0.1573 | 2.4451 | 1.2339 | 0.0152 | 0.1028 | 0.0021 | 3.0745 | 4.5375 | 0.0000 | 0.0110 | 2.5617 | 61.9960 | 15.6394 | 1.6087 | 4.7480 | 0.0000 | 0.0704 | 0.8985 | 0.2257 | 0.0573 | 0.1560 |
| V1-V2 Deg PGM Qiagen Glycerol | 1.1595 | 0.0855 | 2.2869 | 0.0896 | 0.0137 | 14.7729 | 0.0006 | 0.0635 | 0.7142 | 0.0095 | 0.0540 | 13.5469 | 40.9459 | 13.8467 | 1.9990 | 2.8545 | 0.0000 | 0.1158 | 0.6002 | 0.1977 | 1.9740 | 4.6486 |
| V1-V2 Deg PGM RBB Glycerol | 0.9766 | 0.5272 | 2.6250 | 1.0919 | 0.0097 | 13.7369 | 0.0127 | 0.1910 | 1.1182 | 0.0015 | 0.2883 | 17.8065 | 34.5144 | 8.8396 | 8.3543 | 1.0403 | 0.0015 | 0.2981 | 0.7954 | 0.4621 | 1.9352 | 5.3346 |
|  |  |  |  |  |  |  |  |  |  |  |  |  |  |  |  |  |  |  |  |  |  |  |
| V4-V5 Miseq RBB PBS | 0.1668 | 0.0000 | 1.9764 | 10.3054 | 0.0000 | 0.0000 | 3.4779 | 1.1935 | 0.0385 | 0.2053 | 0.0000 | 0.0513 | 25.6289 | 38.5010 | 0.0000 | 10.3696 | 0.0000 | 0.0128 | 0.0128 | 2.0406 | 0.3080 | 1.5529 |
| V4-V5 Miseq Qiagen Glycerol | 0.1114 | 0.0000 | 0.8242 | 8.2201 | 0.0000 | 0.5346 | 9.7349 | 0.0223 | 0.0223 | 0.6906 | 0.2673 | 0.1559 | 2.8291 | 19.2470 | 0.0000 | 0.4010 | 0.0000 | 0.0000 | 0.0446 | 2.3836 | 7.5741 | 45.2885 |
| V4-V5 Miseq RBB Glycerol | 0.1381 | 0.0000 | 1.3538 | 4.9272 | 0.0000 | 0.6907 | 1.7314 | 0.0737 | 0.0000 | 1.3078 | 0.0460 | 0.2026 | 6.0140 | 20.3076 | 0.0000 | 0.5434 | 0.0000 | 0.0000 | 0.0092 | 3.6931 | 6.8889 | 51.3631 |
|  |  |  |  |  |  |  |  |  |  |  |  |  |  |  |  |  |  |  |  |  |  |  |
| V1-V2 Miseq Qiagen PBS | 0.0000 | 0.0072 | 0.8155 | 0.8966 | 0.0000 | 0.0000 | 0.0008 | 0.0273 | 0.0843 | 0.0000 | 0.0000 | 6.5123 | 72.8889 | 14.6211 | 0.0064 | 0.0000 | 0.0000 | 0.3283 | 0.8709 | 0.8444 | 0.0000 | 0.6373 |
| V1-V2 Miseq RBB PBS | 0.0000 | 0.0077 | 0.6127 | 0.3269 | 0.0031 | 0.0000 | 0.0000 | 0.1057 | 0.8276 | 0.0000 | 0.0000 | 4.8960 | 80.2108 | 11.1676 | 0.0081 | 0.0031 | 0.0000 | 0.2107 | 0.3904 | 0.7707 | 0.0004 | 0.3606 |
| V1-V2 Miseq Qiagen Glycerol | 0.0000 | 0.0036 | 0.9363 | 0.9790 | 0.0000 | 0.0187 | 0.0000 | 0.0000 | 0.0463 | 0.0000 | 0.0018 | 25.5761 | 38.2279 | 10.2844 | 0.0018 | 0.0000 | 0.0000 | 0.8268 | 1.1829 | 1.4036 | 0.0036 | 19.9751 |
| V1-V2 Miseq RBB Glycerol | 0.0000 | 0.0207 | 0.7916 | 0.3862 | 0.0000 | 0.1532 | 0.0000 | 0.0128 | 0.3712 | 0.0007 | 0.0000 | 25.0657 | 41.0224 | 9.4461 | 0.0135 | 0.0000 | 0.0014 | 0.8664 | 0.9064 | 1.6595 | 0.0456 | 19.0740 |
|  |  |  |  |  |  |  |  |  |  |  |  |  |  |  |  |  |  |  |  |  |  |  |
| V1-V2 Deg Miseq Qiagen PBS | 0.0000 | 0.0010 | 2.8610 | 1.1073 | 0.0000 | 0.0097 | 0.0000 | 0.0010 | 0.0000 | 0.0000 | 0.0019 | 39.6167 | 0.0107 | 18.6694 | 0.0000 | 0.0000 | 0.0010 | 1.6246 | 2.6291 | 2.5408 | 0.0049 | 30.2766 |
| V1-V2 Deg Miseq RBB PBS | 0.0000 | 0.0000 | 3.6254 | 2.8962 | 0.0000 | 0.0000 | 0.0000 | 0.0531 | 0.0000 | 0.0000 | 0.0000 | 26.9199 | 0.0654 | 56.8076 | 0.0000 | 0.0061 | 0.0000 | 1.2459 | 1.6544 | 4.4322 | 0.0020 | 1.8750 |
| V1-V2 Deg Miseq Qiagen Glycerol | 0.0000 | 0.0000 | 1.4629 | 2.4393 | 0.0000 | 0.0016 | 0.0000 | 0.0000 | 0.0000 | 0.0000 | 0.0016 | 39.4957 | 0.0016 | 14.5357 | 0.0000 | 0.0000 | 0.0000 | 0.8860 | 1.1111 | 2.2554 | 0.0048 | 35.8693 |
| V1-V2 Deg Miseq RBB Glycerol | 0.0000 | 0.0000 | 1.3944 | 1.0328 | 0.0000 | 0.0280 | 0.0000 | 0.0028 | 0.0000 | 0.0000 | 0.0014 | 42.4600 | 0.0028 | 15.0577 | 0.0014 | 0.0000 | 0.0000 | 1.2822 | 1.1996 | 3.7388 | 0.0098 | 33.5291 |
